# The use of multiple evidence base methods to enrich climate change research and knowledge in the Arctic

**DOI:** 10.1007/s13280-024-02093-6

**Published:** 2025-01-25

**Authors:** Máret J. Heatta, Vera Helene Hausner, Tove Aagnes Utsi

**Affiliations:** 1 Department of Sami Teacher Education, Sámi University of Applied Sciences, Hánnoluohkka, 45, 9520 Guovdageaidnu, Norway; 2 Arctic Sustainability Lab, UiT Arctic University of Norway, Framstredet 43, 9019 Tromsø, Norway; 3 Department of Arctic and Marine Biology, UiT Arctic University of Norway, Alta, Norway

**Keywords:** Climate change, Coproduction, Cross-fertilization, Indigenous and local knowledge, Multiple evidence base, Theory of change

## Abstract

Indigenous and local knowledge (ILK) is increasingly used along with scientific knowledge (SK) to understand climate change. The multi evidence base (MEB) offers ways of combining knowledge systems together. Nonetheless, there is little guidance on how to use MEB approaches in research. Our aim is to systematically evaluate empirical cases using MEB approaches in Arctic climate change research; and explore ILK inclusion in research stages. The mapping followed the ROSES protocol, which provides a checklist of details to be included in the review. The literature search identified 1483 records referring to MEB approaches. We identified seven papers applying the cross-fertilization and nine applying the coproduction approach to combine ILK with SK. The theory of change framework was used to evaluate participation, revealing a distinct difference between the approaches in participant involvement in the research stages. Regardless of MEB approach, the output and outcome of the cases were less clear.

**Supplementary Information:**

The online version contains supplementary material available at 10.1007/s13280-024-02093-6.

## Introduction

Indigenous peoples are increasingly engaged with scientists to understand the challenges of environmental change and biodiversity loss and to address the risks of accelerating climate change to society (David-Chavez and Gavin 2018; Lam et al. 2019; 2020; Thompson et al. 2020; Wheeler and Root-Bernstein 2020). Due to their strong relationship with nature and for continuing their way of life, Indigenous peoples are among the people most affected by climate change. Indigenous peoples manage, use, or have land rights for more than a quarter of the world’s land surface area (Garnett et al. 2018), and many spend considerable amounts of their time living of nature as hunters, gatherers, fishers, and herders. Drawing on their experience, knowledge, and information shared in the community, they offer valuable insights into processes in nature, including climate change and biodiversity monitoring and the management of species, land, and seas (Pearce et al. 2015; Eira et al. 2018). The need to include Indigenous and local knowledge (ILK) to understand global environmental challenges and address the dual crisis of climate change and biodiversity loss is therefore also evident in two United Nations panels: the International Panel for Biodiversity and Ecosystem Services (IPBES) and the International Panel for Climate Change (IPCC) (McElwee et al. 2020).

Despite the call to combine ILK along with scientific knowledge (SK) in environmental research and assessments, there is little guidance on how the combining of such widely different knowledge systems, such as ILK and SK, could be conducted. ILK has been defined as the collective knowledge and beliefs rooted in Indigenous and local communities, passed down from generation to generation mainly through oral and practical transmission (Gadgil et al. 1993; Agrawal 1995; Thaman et al. 2013), and is therefore embedded in history, places, and those residing there. The use of ILK as an umbrella term for Indigenous knowledge and local knowledge has been debated among scholars. Both concepts assume a multigenerational association of land and sea that is embedded in cultural beliefs and practices. Those critical of pooling these two knowledge systems into one concept argue that Indigenous knowledge should be defined separately, due to descendance from people that were present before the colonization of another ethnic group and the traditional customs and worldviews that this knowledge is a part of (Hill et al. 2020). In our case, we use the term ILK to reflect, both Indigenous and local knowledge systems with the intent of contributing to a better understanding of the climate and biodiversity challenges that communities are facing in various parts of the world.

ILK has a dynamic dimension where it adjusts with the people as they adapt to the present and the given circumstances (Aikenhead and Ogawa 2007). Combining ILK with SK represents a range of challenges, including the ethical concerns of cherry-picking observations and experiences that specifically answer the questions posed by the research community. Many studies involving ILK are extractive in character with minimal participation in various stages of research, such as problem formulation, design, implementation, analysis, and dissemination (David-Chavez and Gavin 2018; Davis et al. 2021). Environmental observation systems also involve Indigenous communities for data collection, rather than as collaborators for designing, understanding, and interpreting results (Thompson et al. 2020).

Lam et al. (2020) researched how Indigenous peoples have been involved in research on the transformation to more sustainable futures. They found that ILK is primarily used to describe climate change rather than codefining solutions or codeveloping transformative actions for attaining sustainability goals. Wheeler et al. (2020) emphasized that collaborative research involving ILK often poses challenges to Indigenous participants because of historical inequities and power imbalances, and that SK is recognized as a more credible and legitimate source of knowledge than ILK. They point to the need to accept different worldviews, knowledge systems, and ways of understanding the environment to improve research involving ILK.

### Theory

The concepts of braiding or weaving knowledge systems serve as widely utilized metaphors for harmoniously integrating diverse ways of understanding without compromising their individual characteristics and processes (Atalay 2020). At the core of each metaphor lies the principle of preserving the integrity of each knowledge system; while creating opportunities for their distinct insights to converge on shared terrain, thus enabling collaborative solutions to common concerns (Jimmy et al. 2019). Braiding is a relational endeavor that connects various dimensions, such as time, place, values, generations, landscapes, storytelling, and tradition—a process in which everything is interconnected.

Tengö et al. (2014) identified three systematic ways of combining knowledge via multiple evidence base (MEB) approaches: cross-fertilization, integration, and coproduction. These three methods all combine ILK and SK with the purpose of better understanding climate change and addressing climate challenges, but they differ with respect to research practice and degree of validation and synthesis. All MEB approaches involve participatory research to various degrees, which is defined as research that is conducted with and by local people with a focus on locally defined priorities and perspectives (Cornwall and Jewkes 1995). Cross-fertilization is a phenomenon that always occurs when knowledge systems have congruently developed by benefiting from each other (Tengö 2014). Cross-fertilization typically emerges in science-driven projects via methods such as semi-structured interviews and workshops with equal knowledge exchange. This is illustrated in the research of Gordon et al. (2022) on Alaskan recreational and commercial rockfish fisheries. Through cross-fertilization, they bridged harvest data, abundance indices, and management data to gain a more holistic perspective on the ecological status of rockfish. The aim of this method was to create more diversity in the knowledge base through bridging multiple knowledge sources that are more place-based, which can aid the stewardship of fisheries.

Integration resembles the cross-fertilization method, but here, one knowledge system incorporates targeted sources of another knowledge system by comparing or validating information from one knowledge system with the other. ILK has typically been integrated by examining research questions that are ultimately validated via SK. One example is a research project on the impact of increased ship-source noise exposure on cetaceans by Kochanowicz et al. (2021). Inuit knowledge on cetaceans collected by scientists was used in parallel with Western knowledge on cetacean populations together with ship-noise outputs in data modeling. The identification of cetacean areas from the two knowledge systems was compared by measuring overlaps between those two data layers. The integration of SK into ILK could also be a part of this method, with ILK being superior to SK in the integration process.

Knowledge coproduction is a complex concept that expands beyond a method of knowledge creation, it also supports the idea of weaving knowledge systems (Tengö et al. 2017). It is an innovative, adaptable, and introspective process that fosters social learning, builds networks, and informs policy, as well as promoting Indigenous data sovereignty, ownership, and intellectual property rights (Zurba et al. 2022). Knowledge coproduction represents multiple approaches that mitigates power imbalances between disciplines and knowledge systems by avoiding cross-system evaluation and validation; instead, it preserves the integrity of each individual knowledge system. These approaches are anticipated to enhance the relevance and legitimacy of research for Indigenous communities. Moreover, the process compels participants to transcend the history of colonization and collaboratively establish a neutral ground for teamwork through reciprocity (Zurba et al. 2022). Coproduction begins on an equal footing and continues with the exchange and integration of knowledge at various levels and stages, diverging where integration and cross-fertilization occur. Risvoll et al. (2022) researched collaborative and iterative processes with reindeer and sheep pastoralists in various stages of data collection, analysis, and interpretation. The aim of this coproduction of knowledge was to obtain a deeper understanding of the complexity of the pastoralist’s reality in interactions with multiple stressors, which in turn would be used to improve management. Given the different sociopolitical contexts in which knowledge has been produced, we expect the approach of knowledge production to differ depending on the geographical region in question.

Despite the multiple reasons for the benefits of MEB approaches for research and knowledge production, a common consensus on how to carry it out and the effects of different forms of collaborations has yet to be reached. A theory of change (ToC) framework for analyzing the impact of participatory research has been well established after two decades of research (Zurba et al. 2022). ToC was first defined by Weiss (1995) as a methodological tool to analyze “what works” in initiatives to change society. This is especially applicable in coproduction processes with multiple participants participating in the research. The ToC framework main nodes in of a coproduction research process—contexts, inputs, process of implementation, outputs, and outcome—varies greatly across the different research projects and has, to a limited extent, been used to evaluate the use of ILK with SK. In ToC analysis, the main nodes are scrutinized to determine whether they achieve their potential (Connell and Kubisch 1998), this will aid the project directing the actions needed to facilitate transformation and learning from the interventions as it progresses (Rice er al. 2020). According to ToC (Asaaga et al. 2022), there is a greater chance of succeeding with the desired goals if the assumptions of initiatives, such as the MEB approaches, are articulated. The outcomes expected from involving ILK in environmental research should, in such cases, be accompanied by a clear understanding of what and by whom the knowledge is produced, depending on the MEB approach chosen. However, our empirical knowledge about how ILK, along with SK, produces knowledge, the extent of involvement and influence of ILK in environmental research, and the corresponding outcomes depending on approach is limited. Knowledge generation through MEB approaches is at its early conceptual stage, and the reasons for the lack of progress are the lack of understanding of its implementation in different sociopolitical contexts (Zurba et al. 2022), and the complexity and place-based nature of ILK systems. Thus, the MEB approaches that function well under some circumstances are not necessarily transferable to other context. Given this lack of knowledge and the increased desire to include ILK in research, a systematic mapping of the diverse MEB approaches created under different conditions could guide research and policy-makers on how to conduct MEB approaches suitable for different contexts and purposes. Bridging ILK and SK has become increasingly desired in the Arctic due to its potential to improve the quality and relevance of climate change research, and to ensure community engagement in addressing the climate-related challenges that the Arctic residents are facing (Tengo et al. 2014; Brattland et al. 2019; Kettle 2019; Curry and Lopez 2020; Norström et al. 2020).

The objective of this review is to systematically map empirical cases that use the MEB theory presented by Tengö et al. (2014) to combine ILK and SK in the production of climate change knowledge in the Arctic. Drawing on the different MEB approaches to knowledge production, we ask: how do MEB approaches in climate change and environmental research vary across the Arctic region? Who is involved and in which stages? Finally, by using the theory of change framework, we also examine the potential outcomes or impacts of research on knowledge generation related to rapid climate change in the Arctic. We will in this paper systematically map empirical cases that use an MEB approach by combining ILK and SK to understand climate change in the Arctic. A systematic map of empirical research is beneficial for evaluating where, how, with whom and in which sociopolitical context the knowledge was produced to evaluate the outcome and create pointers for future research. MEB approaches in the Arctic are particularly interesting given the emphasis on bridging ILK with SK in the Arctic Council. The Arctic Council is an intergovernmental forum that was established in 1996 and has grown to become an important platform for collaboration among Arctic countries, particularly those related to environmental monitoring and sustainable development. This emphasis is expected to continue, even though it is facing challenges in the absence of Russia (Jonassen 2022). The North American Arctic is emerging as a key area for implementing numerous MEB approaches related to climate change with Indigenous peoples onboard or in charge (Davis et al. 2021). This includes more funding to Indigenous-led research as well as more research agendas led and designed by Indigenous peoples.

## Methods

There is a rich body of literature examining the collaboration between Indigenous communities and Western scientific practices. Here, we study how collaboration occurs and contributes to understanding the effects of climate change in the Arctic. Our focus lies in the realm of research and education within the natural sciences, with a keen emphasis on sustainability and the integration of ILK. Each member of our team has extensive experience living and working in various regions of the Arctic, engaging with diverse Indigenous research initiatives. Notably, the first and second authors of this study are of Sami descent. The first author resides in the Sami village of Guovdageaidnu, Norway, where Sami is her primary language. Author two, with Sami heritage and Norwegian as her primary language, resides in the city of Romsa, Norway. Author three, Norwegian by birth, is married to a Sami reindeer herding family in Finnmark, Norway, and resides near the city of Áltá, Norway. Our approach to Indigenous matters is founded on a sincere desire to contribute positively to ongoing discussions. Given that our study is a review, encompassing no new empirical data, we have not deemed it necessary to seek feedback from additional Indigenous peoples.

We searched the peer-review and gray literature referring to MEB approaches. We opted to compare the different MEB approaches delineated in Tengö's publication. Given the extensive body of literature characterizing these concepts in various ways, our selection of papers encompasses a consistency in definitions of the terms. We created a protocol based on the reporting standards for systematic evidence syntheses (ROSES) in environmental research. The mapping was performed in consecutive stages designed to answer the research questions. The method included five stages: setting the scope and question, searching for papers, screening papers, coding, and visualizing; and describing the findings. The search stage was performed via two web-based search engines, the ISI Web of Science and Google scholar. Google Scholar is particularly relevant for searching for gray literature, whereas the ISI Web of Science is a specialized database for peer-reviewed articles.

**Fig. 1:**
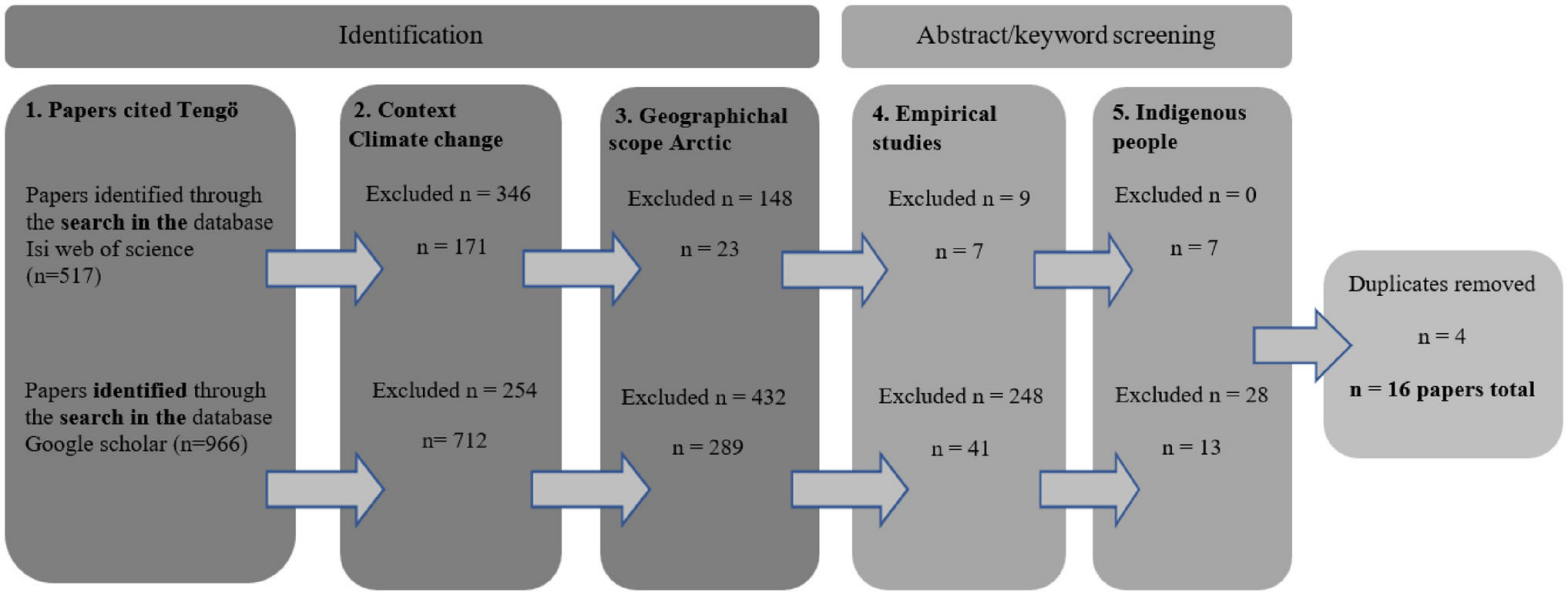
Flowchart, showing the process for identifying the studies that were relevant for this analysis. The literature published between March 2014 and June 2023 were included

The search was reviewed in two rounds: paper identification and abstract plus keyword screening (Fig. 1). The first round of selection was conducted in three stages. The first stage included the identification of peer-reviewed journal articles that referenced Tengö et al. (2014) published and/or accepted between 2014 and June 2023, when we decided to stop the search. We then excluded articles that were not related to climate change or the Arctic. We applied the search words “climate change” in the abstract, and key words among the results for the referenced Tengö et al. (2014) papers. There is no single agreed upon definition of the Arctic, but for this purpose, we used the AMAP definition, and its coverage area, which is: “*the terrestrial and marine areas north of the Arctic Circle (66°32'N), and north of 62°N in Asia and 60°N in North America, modified to include the marine areas north of the Aleutian chain, Hudson Bay, and parts of the North Atlantic Ocean including the Labrador Sea.” * (AMAP 1998). The criteria “empirical studies” and “Indigenous peoples of the Arctic” were implemented by screening titles and abstracts. For this we used a list of the recognized Indigenous peoples of the Arctic created by Arctic Indigenous Peoples Languages and Revitalization (2023) (search string Indigenous people and table 10.1007/s13280-024-02093-6 first round of the article selection process in Supplementary Information). Duplicates were excluded at this stage.

Following full-text screening, the papers were extracted via the NVIVO 1.7.1 software package for qualitative data analysis. Data charting was completed with a focus on title, author(s), year, study methods, study objectives, country, climate change impact, input, output, and outcome. All the results were downloaded as reference files to End Note X9.3.3 desktop version. The classification of MEB approaches was performed through thematic content analysis of the methods used in the individual studies to determine whether they qualify for cross-fertilization or coproduction. Thematic content analysis is a method used in qualitative research to analyze textual data and identify themes (Vaismoradi et al. 2016). We applied the ToC framework to evaluate the research inputs, outputs, and outcome in the empirical case studies. The ToC framework shows how various activities and resources lead to immediate results, which in turn lead to short terms and long-term changes.

Participants in a research project can be described as any person with a stake in the research theme through volunteer engagement or who received an invitation to contribute key knowledge (Tengö et al. 2021). The participants and the knowledge they contribute to the research are categorized under the input node in the ToC framework. The participants in the studies described in the papers analyzed were grouped into scientists, Indigenous scientists, and a finer categorization was made for Indigenous and non-Indigenous participants related to their role in their community. If indigeneity was not explicitly stated in the paper, they were categorized as other participants or scientists. The coproduction processes were distinguished by involving participants at an early stage in the process, either at the research initiative or at the development of research questions (involvement > 50% of stages). Cross-fertilization was characterized by the involvement of fewer steps and a later stage, typically when the project had already been established by researchers.

The objectives of the different research team projects were classified according to the aim of the study and the problem that the team desired to address (Table 1). The classifications were iteratively defined on the basis of definitions created by intergovernmental/international environmental authorities such as the United Nations Environment Program (UNEP) and IPCC. For objectives that presented new ways of researching or generating knowledge about climate change, we created the category *Novel methods and tools* (Table 1). This category included research that attempts to design tools to improve the relevance and quality of climate research.

**Table 1:** The categories of objectives described in the studies and their definitions. The categories are based on the definitions of International Panel for Climate Change (IPCC) and United Nations Environment Program (UNEP) with some changes, in addition to the own defined category; new methods and tools

Category of objectives	Definition
Climate impact	Study of the effects of climate change on ecosystems, resources, traditional practices, and how it impacts the people that depend and value them” (IPCC).
Novel methods and tools	Design and implementation of methods and tools to improve relevance and quality of research, and/or management of ecosystems where Indigenous and local people inhabit.
Climate risk perception/climate perception	Climate change will lead to numerous risks to the human and natural world. Climate risk perceptions are people’s knowledge, beliefs, attitudes, responses, and concerns to climate change consequences and likelihood (IPCC).
Empowering and capacity building	Enhance the capacity of the targeted community/study area/people to take control over their own development/situation in research, management, climate change mitigation and adaptation, and to develop strategies and address them (IPCC).
Ecosystem-based adaptation	Defined by UNEP as approaches that create ways based on the local ecosystems and biodiversity to help people adapt to the impacts of climate change. These nature/based approaches built on Indigenous and Local Knoweldge (ILK) and/or people’s observations have the potential to raise climate change awareness, resilience as well as protect the natural environment.

**Table 2:** Studies in the coproduction approach categorized in the distinct categories of objectives

Categories	Studies	Output
Empowerment and capacity building	Falardeau et al. (2018)	Pockets of past—scenario model for climate change to design appropriate adaptations and approaches to the future
	Risvoll et al. (2022)	Provides status on the mismatching interlinkages of pastoralism and national policies in animal husbandry governance.
	Horstkotte et al. (2017)	Documentation and empowerment of reindeer herder knowledge on climate change.
Novel methods and tools	Curry and Lopez (2020)	A model with visual images to improve communication between place/based information beyond potential of basic text that can be beneficial for management.
	Peacock et al. (2022)	Indicator-based assessment framework with a traffic light approach to connect information from separate monitoring initiatives.
Ecosystem-based adaptation	Burt et al. (2020)	Learning platform to expand the knowledge about sea otter and the traditional values tied to the resource, which can foster successful future co-management efforts.
	Gérin-Lajoie et al. (2018)	Community Based Monitoring (CBM) framework with recommendations for local success.
Climate change impacts	Gryba et al. (2021)	Documented information on spatiotemporal scale about habitat use, foraging activity, and dive behavior of bearded, ringed, and spotted seals.
	Falardeau et al. (2022)	Climate impact approach designed by locals that bridges different pieces of evidence into a cohesive and nuanced story of ecological changes and their socio–ecological implications.

The output was identified through thematic content analysis as either a tangible or intangible product of the research. We did not expect many studies to report on the outcome as it refers to the impacts of the project usually occurring after publishing the paper, but we analyzed the researchers’ own reflections on the implications of their study relating to their objectives.

## Results

The literature search resulted in total of 1483 papers. After removing duplicates, nonempirical studies, books, book chapters, review papers, proceedings, reports, and papers not written in English, a total of 16 papers remained which were categorized in terms of MEB approaches (Fig. 1). These were categorized into the approaches according to the degree of participant involvement. The papers were nearly to evenly distributed into categories; seven papers in the category of cross-fertilization and nine papers in the coproduction category. The third category, integration, was not found in the analysis of the articles as there were no research projects that clearly met the criteria. The papers were geographically distributed unevenly, with most of the papers published from Canada (6), followed by Alaska (4), Sápmi (3), and Russia (2); one paper was a collaboration between Alaska and Canada (British Columbia).

### Participant involvement and data collection in the studies

**Fig. 2:**
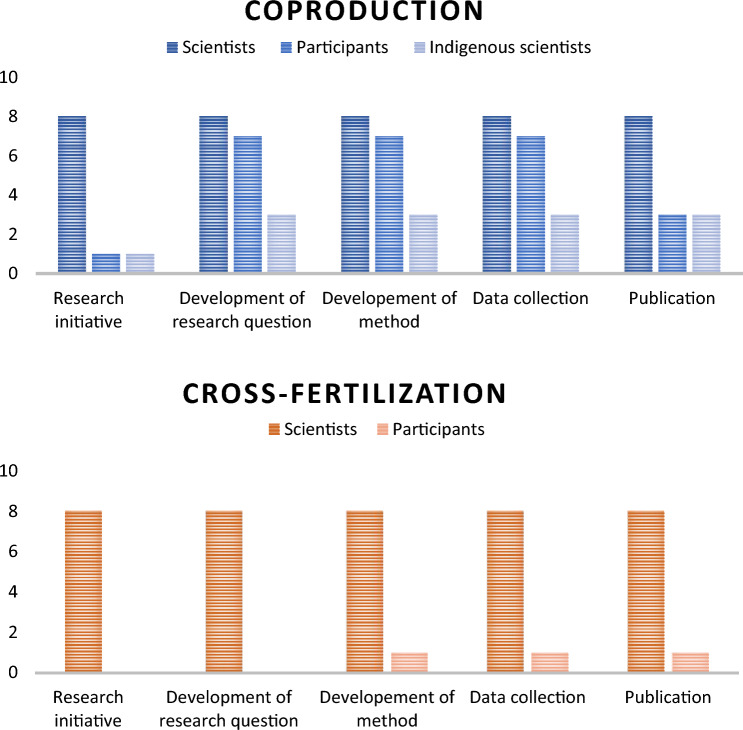
The research stages and the involvement of scientists and participants at each stage are presented separately for the two Multiple Evidence Base (MEB) approaches

**Fig. 3:**
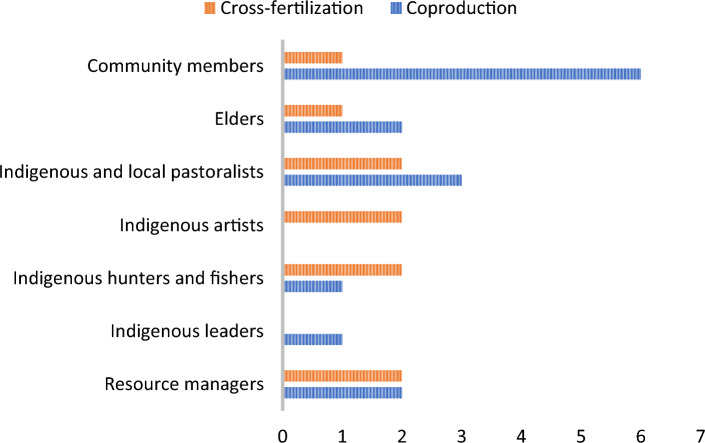
The different participants participating in cross-fertilization versus coproduction projects. We categorized the various participants reported in the studies according to the resemblance of their practices, occupation, and belonging. «Indigenous artist “where crafters and painters. The participant groups not marked with Indigenous indicate that the group could consists of both Indigenous and non-Indigenous members as described in the studies

**Fig. 4:**
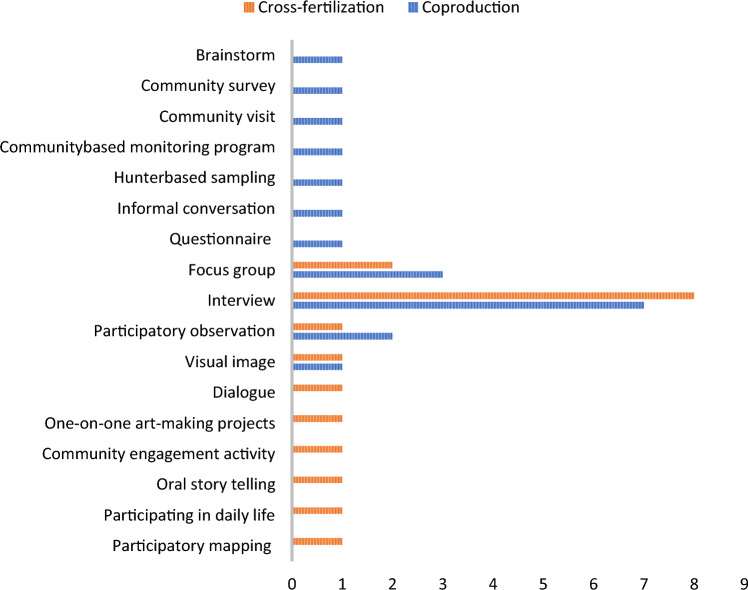
The participatory research methods in the different studies divided into the two Multiple Evidence Base (MEB) approaches, cross-fertilization and coproduction. The most common methods in both approaches were interviews

The analysis of participant involvement in the various stages revealed the clearest difference between coproduction and cross-fertilization (Fig. 2). In coproduction research, the participants were involved in the development of research objectives and continued to participate in the distinct stages, with the exception of authoring papers as only four publications were coauthored with nonscientists. Most of the projects were initiated by Western scientists. Three of the papers were initiated by Indigenous scientists or researchers previously associated with the community that engaged in the study. In addition to scientists, the majority of the participants were Indigenous or living in Indigenous communities. The participants were divided into MEB approaches according to the study from which they came (Fig. 3). The participants in the cross-fertilization category were mostly “Indigenous artists,” “Indigenous hunter and fishers,” “pastoralists” and “resource managers,” whereas in the category of coproduction the participants represented a diverse range of community members, including youth. In both the cross-fertilization and coproduction cases, the most common methods were interviews (Fig. 4). This is also the most common research method in qualitative research, generally (Cassell 2005). The interviews were primarily semi-structured interviews, whereas a few studies used informal interviews, survey interviews, and unstructured interviews. The interviews were conducted solely by the scientists in both MEB approaches. Additionally focus groups and participatory observations were frequently used in both categories. In papers categorized under coproduction, a total of 34 different methods were utilized, indicating a slightly greater diversity in the research methods of choice compared to the papers categorized under cross-fertilization, which used 29.

### Objectives and output

The objectives of the studies were classified into five categories: *Novel methods and tools* had five cases, followed by the categories *Climate risk perceptions*, *Empowerment and capacity building*, *Climate impacts*, and *Ecosystem-based adaptation*, which had two cases each. After grouping them into the two MEB approaches, we observed greater diversity in the objective categories in the coproduction approach (Table 2) than in the cross-fertilization approach (Table 3). *Novel methods and tools* included of studies with research interventions, such as how traditional artwork can be used to illustrate and gain insights into climate change, as well as convey information beyond the potential of written text for management purposes (Curry and Lopez 2020; Rathwell 2020). Peacock et al. (2022) incorporates local ecological knowledge (LEK) and scientific wildlife monitoring methods with a traffic light approach to evaluate the conservation status of species. This method is based on red light signified poor population health, necessitating high levels of management intervention. Green light signified good population health, requiring low level management intervention. The color amber fell between these two extremes. By combining these diverse sources of information, researchers can make more informed decisions about conservation strategies and prioritize effective management actions. Studies in the category *Climate risk perception* addressed issues on how rapid climate change and an increasing human footprint are threatening the health of Arctic wildlife and Indigenous communities, as well as changes in the sea ice condition such as timing of sea ice formation and loss, thinning of ice, spatial extents, ice texture, land-fast ice breaking up earlier than before and loose pack ice remaining in the area. The papers reported changes in floral and faunal species composition across the Arctic. In the objective category *Empowerment and capacity building*, pastoralists were given a voice in research about their experiences with vegetation change in pastoral landscapes, such as the increased abundance of shrubs and the progression of tree lines (Horstkotte et al. 2017) and predator increases (Risvoll et al. 2022). In the objective category *Climate impacts*, Falardeau et al. (2018) documented Inuit fishers’ observations of change in the migration patterns of the Arctic char (*Salvelinus* *alpinus*) and the timing of migrations and the length of their marine feeding season. Additionally, invading boreal apex predators’ interaction with Arctic Char through competition and predation. The research project conducted by Gérin-Lajoie et al. (2018) falls under the objective category of ecosystem-based management. The study aimed to collaboratively design a Community-Based Monitoring (CBM) program with local people in Nunavik, Quebec. The primary goals were to enhance resource and water quality within a significant watershed and ensure the transmission of ILK.

**Table 3:** Studies in the cross-fertilization approach categorized in the distinct categories of objectives

Categories	Studies	Output
Novel methods and tools	Mellegård and Boonstra (2020)	Reconnection of people to nature and thereby facilitate sustainable development.
	Rathwell (2020)	Platform with artworks and artistic processes to connect with the many dimensions of knowledge, including content, values and beliefs, emotions, and sensory experience.
	Kochanowicz et al. (2021)	Identification of cetacean populated areas by complementing Inuit knowledge and western scientific knowledge to support decision- and policy-makers in their plans to manage vessels in Tallurutiup Imanga National Marine Conservation Area, Canada.
Ecosystem-based adaptation	Gordon et al. (2022)	Model with multiple knowledge forms to generate a collective understanding of rockfish populations and fisheries for place-based, community-driven stewardship.
Climate risk perceptions	Crate (2021)	Knowledge documentation.
	Crate (2022)	Description of the multifaceted traditional Indigenous land and the treats climate change imposes.
	Luizza et al. (2016)	Integrative model for invasive species with concerns of communities affected incorporated for holistic risk assessment and more inclusive and collaborative management.

### Output and outcome

Within the papers examined in this review, we identified various outcomes, including the development of models for biodiversity monitoring and the design of effective strategies for climate change adaptation tailored to local communities (Luizza et al. 2016; Falardeau 2018; Peacock et. al 2022), or immediate benefits such as increased trust, improved communication, and relevancy of research and policy through bringing together a diversity of participants in research (Kochanowicz et al. 2021; Crate 2022; Gordon et al. 2022), as well as facilitating knowledge transmission across generations in community-based projects (Gérin-Lajoie et al. 2018). As ILK is on the verge of disappearing with new ways of life, Horstkotte et al. (2017) and Crate (2021) emphasized the importance of documenting ILK for empowerment in management purposes. Both Curry and Lopez (2020) and Rathwell (2020) created a communication platform with art and craftwork as boundary objects to convey ILK and local perceptions of climate change inside the participant group during a research project and later to policy-makers. Similar platforms have been created for other purposes, such as social learning (Burt et al. 2020). Risvoll et al. (2022) described the mismatching interlinkages of pastoralism and national policies. A consistent output throughout the cases in this review was ILK documentation for various purposes such as local climate change perceptions in leu of cultural framing (Crate 2021) or providing research on a finer spatiotemporal scale than scientific methods (Gryba et al. 2021) (Table 2).

The outcome, as defined in the ToC framework, is the impact the research has or will have for the purpose of the project. Given the need for a long-term perspective to assess research impact, none of the papers reported immediate outcomes upon publication. However, nearly all of these studies highlighted potential outcomes. These included enhanced cultural understanding among participants unfamiliar with the local culture, (Gérin-Lajoie et al. 2018; Rathwell 2020), and climate change models designed with an MEB approach, creating suitable adaptation strategies for future climate change scenarios both for community and nature management (Luizza et al. 2016; Falardeau et al. 2018; Gryba et al. 2021). Many projects reported that their effort in bridging different knowledge systems and data sampling methods will contribute to a management policy that is more comprehensive, holistic, ethical, empowering, and locally relevant.

**Fig. 5:**
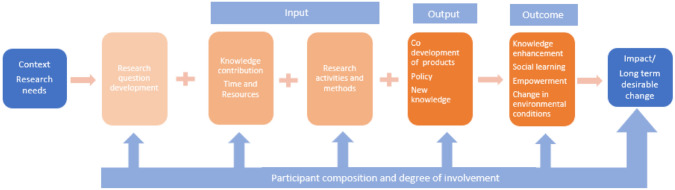
The Theory of Change (ToC) nodes and their respective content summarizing findings in the systematic mapping

Figure 5 illustrates the nodes and their respective content, summarizing the findings reported in this mapping review. Context is the environment, including institutional and cultural factors, where the research is taking place. The inputs are all the resources and activities put into a project to make it happen (e.g., the knowledge of the Indigenous, local and scientific participants in the project, funding or time put into the research, data sampling). The outputs are the results generated by the research project in which the research participants are given access to or benefit from tangible products such as artwork, knowledge documentation, etc., or non-tangible products such as a variety of models, policy, and new knowledge. However, the outcome is the longer-term impact that the research has or will have on the long-term impact of the project, such as empowerment, knowledge enhancement, and social learning.

## Discussion

The papers selected in this mapping show the diverse ways in which research has combined ILK and SK in climate change research. The papers revealed an uneven geographical distribution of research projects utilizing MEB approaches. There has been significantly more research from the North American Arctic, which was also discovered by Davis et al. (2021). This might indicate that knowledge production through novel methods such as cross-fertilization and coproduction is more desirable in these areas because of the history of colonization of Indigenous peoples and land and resource disputes (Dowie 2011). The large spatial distribution of Indigenous land in the Arctic (Garnett et al. 2018) and its remoteness may have caused a need for local participation in research, especially on Indigenous land.

Early forms of participatory research emerged in North America in the middle of the twentieth century in response to power disparities and injustices in research at the time, and Indigenous peoples expressed fatigue with repeated “parachute science,” when researchers played a significant role in the planning and execution of the study (Macaulay 2016). In the last two decades, there has been a rapid increase in more socially just research methods across a number of disciplines (Brown 2022), encouraged by research funding (Wallerstein 2017). An inquiry about more differentiated research methods as we have highlighted in this review, as well as the inclusion of diverse knowledge systems to steer and lead the research, was presented. This is transferable to the decolonization of research, which characterizes an ongoing process in Indigenous research. The term refers to a process of research that replaces dominant Western research paradigms imposed by colonialism, with engaging methods in which Indigenous epistemologies and ontologies mirror the research process (Smith 1999; Tuck and Yang 2012; Thambinathan and Kinsella 2021). In Sápmi, despite power imbalances still in place (Eriksen et al. 2021; Kater 2022), the decolonization of research was well underway even before the concept of Indigenous methodologies emerged in the late 1990s (Porsanger and Seurujärvi-Kari 2021). Sami people claimed their seat in politics and academics and successively established institutions for self-governed research already 20 years prior (Virtanen et al. 2021). This may have influenced the research over time, which is evident among the papers in this review, where in Sápmi, the researchers either have close relationships with the Indigenous peoples or Indigenous scientists.

In this mapping, we focused on the categories cross-fertilization and coproduction, as there were no papers that clearly fell under the category Integration. Integration has become the scapegoat in knowledge generation, as it is a method where knowledge goes through a validation process, where scientists choose bits and pieces from Indigenous knowledge that conveniently meet research needs (Ludwig 2016). Notably, none of these papers chose the method of integration, indicating that the focus of knowledge generation is moving toward a direction where knowledge holders are given a voice throughout the research process. The participants have in such cases the opportunity to express concerns and to provide input (Salomon et al. 2018). A broader engagement in the research process could also enhance the mutual understanding of goals and community culture (Meyer et al 2022).

We found no substantial differences in the two MEB approaches, cross-fertilization and coproduction, in terms of the various parameters of the ToC framework. However, both of these approaches are participatory research method, with a larger number of people with diverse backgrounds, than traditional scientific research. Coproduction implies that participants are included at a much earlier stage, and they participate throughout all stages of the research process, compared with cross-fertilization, in which participation may occur at a later stage or cease before closure. This is in line with Davis et al. (2021), demonstrating that the degree of participation varied greatly throughout the research projects, with the majority reporting local participation in the first stages of the research; participant identification—and the data generation process. Low participation was reported in the output generation and in the evaluation of the projects. Coproduction implies that participants are included at a much earlier stage and that they participate throughout all stages of the research process. Nonetheless, both contribute to democratizing research, and are in line with the trends seen in climate change research with research focused on local adaptations with local participation throughout the project, rather than only relying on quantitatively obtained climate projections or impacts (Bhave et al. 2014; Conway et al. 2019).

Compared with cross-fertilization, the coproduction approach demonstrated slightly greater number of data sampling methods. This could be attributed to the participants' increased autonomy in codesigning the research process via this approach. Altogether, the methods of data collection for both approaches are diverse and can range far from the expected methods of traditional scientific research in this field, as shown in this selection of papers. The creativity of the choice of method in the various projects reflects the need for a project to exert a certain amount of plasticity when collaborating with the different participant groups, places, and knowledge available. This is a demanding process, with both pitfalls and possibilities (Howarth et al 2022). Falardeau et al. (2018) argued that in addition to bridging different knowledge systems to learn more about climate change impacts, coproduction also increases trust and fosters a sense of ownership of the project, allowing results to be implemented by the community. The reason for the small number of coproduction studies can be traced to the actual costs and logistical challenges of the method, but ultimately it may outweigh some of the pitfalls of external initiated and produced research projects. In their coproduction project, Falardeau et al. (2018) observed in their coproduction project community members expressing a wish to be heard and participate in research, as opposed to centuries of neglect. Giving voices to communities experiencing climate change first-hand creates opportunities for the delivery of relevant and impactful outcomes. Climate change clearly has major implications for Indigenous communities in the Arctic, the impacts observed are ample and complex, and little is known about what the future will bring. To develop culturally acceptable and actionable adaptation strategies, it is crucial to understand local situations and perceptions. For example, local reindeer herders’ observations offer valuable insights into local conditions, extreme weather events, and their effects on livelihoods (Rasmus et al. 2020).

The results presented here have limitations in terms of scope and depth. First, the chosen search criteria are subject to limitations. We focused primary on the studies referenced Tengö et al. (2014) and studies with comparable research designs were excluded. Expanding the search criteria could enable the inclusion of additional studies and possibly even out geographical bias. Second, the two approaches, coproduction and cross-fertilization, were separated mainly according to the five stages of the research projects in which the participants were engaged, or the group participating in the projects, in addition to how many stages of participation. The separation process was performed through an analysis of the methods section in the paper. There was a general lack of reported method details in the papers; we encountered that the participation and participants were underreported, or not highlighted enough, which may have caused some studies to be miscategorized. There was also an absence of reported participant experiences that reflects the MEB methods utilized in all studies. Aspects, such as ways of social learning, trust-building, and partnerships, were not fully reported in the papers. Including this may help direct future research into choosing methods that are truly democratizing in all senses.

Participatory research has become a common method in climate change research and mitigation planning, such as the coproduction and cross-fertilization approaches discussed in this paper (Davis et al. 2021). In a systematic mapping paper, the outcome is not measurable owing the timeline of the publication process. Most of these papers predict and hope that the respective outputs will be beneficial and potentially contribute to future climate adaptation by being more relevant and place-based, relying on local voices and potentially marking a starting point for a long-term vehicle that will produce the expected outcomes over time (Reed et al. 2023). The outcome of participatory research, which includes participants from a wide range of interests, will naturally be multileveled, including altered trust in researchers and inclusion independently experienced by various participants. While addressing climate change can be challenging at the local level, it necessitates perceiving it as a direct threat or a problem affecting our daily lives. However, climate change is inherently complex and nonlinear, with gradual, often invisible effects. Many people experience these effects at a distance from their immediate surroundings, making personal connection and motivation for action difficult. This challenge may also influence research project outcomes.

The recommendation for future research is a more explicit focus on the results and outcomes of the different approaches used to identify benefits at the community level. This may provide a deeper comprehension of climate change and its possible effects on Arctic ecological and societal systems. In addition, future studies should go beyond reviewed literature to analyze the impact of participation in the research and the inclusion of ILK as well as the knowledge produced.

## Conclusion

We found no substantial differences between the two MEB approaches in this study, but both cross-fertilization and coproduction are likely to produce relevant arenas to convey knowledge between communities and policy-makers, and to make relevant climate change adaptation plans. We found relatively few empirical cases that utilized coproduction and cross-fertilization, and the large majority were located in North America, for reasons that are likely to be historical. In our analysis, we also found a slightly greater diversity of participants and a greater variety of methods applied in the coproduction approach. There was also a slight tendency for the coproduction approach to involve participants earlier in the research process, in line with the definition of the approach. The cultural gap that many Indigenous peoples experience when dealing with non-Indigenous scientists often gets in the way of a mutual understanding of climate change and its impacts; however, when MEB techniques are used in research, this gap may lessen or close. Further suggestions and directions for users of coproduced knowledge and policy-makers include the importance of early stakeholder inclusion and engagement through participatory processes with diverse stakeholder involvement. It would be beneficial for all participants' outcomes if local participants were further encouraged to learn and develop their capacities. In addition, establishing safe and transparent ethical principles and acknowledging the rights of Indigenous peoples to govern and manage their knowledge are critical.

In the future, there is a need for follow-up studies that could track the impact of MEB approaches, but it would also benefit coproduction and cross-fertilization processes, if researchers who conduct these studies, were clearer about the results and impacts expected from their research. Future research should prioritize developing the current position of ILK toward more equity in research and mitigation and adaptation planning, especially as the effects of climate change affect people’s ways of life and may cause the loss of ILK.

## Supplementary Information

Below is the link to the electronic supplementary material.

Supplementary file1 (PDF 1058 KB)
