# Supplementary material for: The use of multiple evidence base methods to enrich climate change research and knowledge in the Arctic
Source: Ambio. 2025 Jan 25;54(4):603–17. doi: 10.1007/s13280-024-02093-6 (PMC11871264; doi:10.1007/s13280-024-02093-6)
Supplement: Supplementary file 1 — Supplementary file1 (PDF 1058 KB) [file 13280_2024_2093_MOESM1_ESM.pdf]

AMBIO
Supplementary Information

# Title: The use of multiple base evidence methods to enrich climate change research and knowledge in the Arctic

## Appendix S1

### Search String Indigenous people

"Alaskan Athabaskans" OR "Aleut" OR "Aleut" OR "Aleut language family" OR "Aleutian" OR "Aleuts" OR "Aleuts" OR "Alyutor" OR "Alyutors" OR "Chelkan" OR "Chelkans" OR "Chugach Sugpiaq" OR "Chukchee" OR "Chukchis" OR “Chukchi” OR "Chulym" OR "Chulyms" OR "Chuvan" OR "Chuvans" OR "Cup'ik" OR "Deg Hit’an" OR "Dena'ina" OR "Dolgan" OR "Dolgans" OR "Enet" OR "Enets" OR "Entsy" OR "Eskimo" OR "Evenk" OR "Evenki" OR "Evenks" OR "Eyak" OR "Faroese" OR "Fort McPherson" OR "Fox Islanders " OR "Greenlander" OR "Greenlandic language" OR "Gwich’in" OR "Gwichin" OR "Gwitchin" OR "Haida" OR "Holikachuk" OR "Hän Hwëch'in" OR "Icelander" OR "Inuinnaqtun" OR "Inuit" OR "Inuit" OR "Inuit Tapiriit Kanatami" OR "Inuits" OR "inuk" OR "Inuktitut" OR "Inuktun" OR "Inupiat" OR "Iñupiat, an Inuit group" OR "Inuvialuit" OR "Inuvialuktun" OR "Inuvik" OR "Itelmen" OR "Itel'men" OR "Itelmens" OR "Kalaallisut" OR "Kamchadal" OR "Kamchadals" OR "Kerek" OR "Kereks" OR "Kety" OR "Khant" OR "Khanty" OR "Kitikmeot" OR "Kivalliq" OR "Koniag Alutiiq" OR "Koryak" OR "Koryaks" OR "Koyukon" OR "Kumandins" OR "Laplanders" OR "Lapp" OR "Lapps" OR "Lopars" OR "Makivik" OR "Mansi" OR "Nanai" OR "Negidals" OR "Nenets" OR "Nenetsy" OR "Nentsy" OR "Nganasan" OR "Nganasans" OR "Nivhgu" OR "Nivkhi" OR "Nivkhs" OR "Northwest Territories" OR "Nunatsiavut" OR "Nunavut Tunngavik" OR "Old Crow (Vuntut Gwitchin First Nation)" OR "Oroch" OR "Oroch people" OR "Orochi" OR "Orok" OR "Oroki" OR "Oroks" OR "Ostyak" OR "Ostyaks" OR "Pauktuutit" OR "Qawalangin Tribe" OR "Qikiqtani" OR "Saami" OR "Sakha" OR "Samalga" OR "Sami" OR "Samoyed" OR "Samoyeds" OR "Sapmi" OR "Selkup" OR "Sel'kup" OR "Selkups" OR "Siberian Yupik" OR "Soyots" OR "Sugpiaq ~ Alutiiq" OR "Tanacross" OR "Tavgi" OR "Telengit" OR "Telengits" OR "Teleut" OR "Teleuts" OR "Tlingit" OR "Tofa" OR "Tofalar" OR "Tofalars" OR "Tozhu" OR "Tsiigehtchic" OR "Tsimshian" OR "Tubalar" OR "Tubalars" OR "Tunumiit" OR "Udege" OR "Ulch" OR "Ulchs" OR "Umnak" OR "Unalaska" OR "Unangan" OR "Unangax̂" OR "Upper Kuskokwim (Kolchan)" OR "Upper Tanana"  OR "Vogul" OR "Voguls" OR "Yakut" OR "Yenets" OR "Yenisei Ostyak" OR "Yenisei Samoyeds" OR "Yukaghir" OR "Yukaghirs" OR "Yukagir" OR "Yukagirs" OR "Yukon" OR "Yupik" OR "Yup'ik"

# Table S1 First round of the article selection process

| | Search terms | ISI Web of Science | Excluded | Google scholar | Excluded |
|---|---|---|---|---|---|
| | | **1. Round: Identification** | | | |
| 1.stage<br>**Method** | **Cited Tengö (2014)**<br>Method MEB<br>Peer reviewed papers | 517 | | 966 | |
| 2.Stage<br>**Context** | “Climate change” | 171 | 346 | 712 | 254 |
| 3.Stage<br>**Geographical scope** | Arctic | 23 | 148 | 289 | 432 |
| | | **2. Round: Abstract/keyword screening** | | | |
| **Empirical studies** | | 7 | 9 | 41 | 248 |
| **Indigenous people in the Arctic** | Searchstring indigenous people ("Alaskan Athabaskans" OR "Aleut" OR "Aleut" OR…..? | 7 | 0 | 13 | 28 |
| **Duplicates:** | | | 4 | | |
| **Total** | | | **16** | | |

# Table of papers examining with theory of change

| | | | | | Method/Input | | | | Involvement/Input | | | | | Output |
|---|---|---|---|---|---|---|---|---|---|---|---|---|---|---|
| | **Knowledge bearers** | **MEB approach** | **Objectives** | **Categories of objectives** | **Participant consultations** | **Quantitative methods &scientific methods** | **Participatory methods/ qualitative** | **Data - member checked** | **Research initiative** | **Development of research question** | **Method development** | **Data collection** | **Publication** | |
| 1. | ILK experts (elders), managers at a local and federal organization | Co-production | Create a guide for sustainable action for people to future environmental and social change through mobilizing ILK | Empowerment and capacity building | Interviews, community surveys, focus groups, meetings with the local hunters and trappers' organization | - | Workshop | Participants involvement throughout the process | Scientists | Participants along with scientists | Participants along with scientists | Participants along with scientist | Scientists | Advancing the method for mobilizing ILK in participatory knowledge production for this particular project, as well as to inform other. Brought together a diversity of stakeholders to explore and plan around the future. Scenario model produced for climate change to design appropriate adaptations and approaches to the future. Transferable to other settings for building local participatory scenarios |
| 2. | Fisher, duojár | Cross - fertilization | Explore how local natural resource users' knowledge can be methodologically identified and theoretically articulated for sustainable use | Novel methods and tools | Not reported | - | Participatory observation, interviewing and photography | Yes | Scientists | Scientists | Scientists | Scientists | Scientists | Craftmanship can reconnect people to nature and thereby facilitate sustainable development. More knowledge is needed on how this is possible. |
| 3. | Resident adult population, hereditary chiefs, community leaders, community members | Co-production | (a) What social-ecological conditions are perceived to enable people's ability to adapt to sea otter recovery, and which are most enabling? (b) How do perceptions of these conditions differ between communities? and (c) How variable are people's attitudes towards sea otters and what factors might influence this? | Ecosystem based adaptation | Collaborative research partnership with stakeholder organizations | Literature search – grounded and resilience theory | Workshop, focus groups, survey interviews and community visit | Co-production protocol with members participating in all stages of research. | Scientists | Scientists and indigenous stakeholders | Scientists and indigenous stakeholders | Scientists and indigenous stakeholders | Scientists and indigenous stakeholders | The project created learning platform to exchange management outcomes through gathering across stakeholders and management. This is reported to expand the knowledge about sea otter and recover and the values tied to these resources for all parts that can foster successful co-management efforts. |

| | | | | | | | | | | | | | | |
|---|---|---|---|---|---|---|---|---|---|---|---|---|---|---|
| 4. | Reindeer herders and sheep farmers | Co-production | how consequences of multiple drivers of change and fragmented governance affect adaptation and subsequently long-term sustainability of Nordic pastoralism. | Empowerment and capacity building | Conversations, interviews, and participatory observations | Written sources such as policy documents, peer-reviewed gray literature | Semi structured interviews, informal conversation, participant observations | Yes, defined research questions together with herders and farmers and continued an iterative dialogue throughout the analysis and preparation of this article. | Scientists and participants | Scientists and participants | Scientists and participants | Scientists and participants | Scientists | Provides status on the mismatching interlinkages of pastoralism and national policies. Study shows that radical change is needed toward a more holistic animal husbandry governance where multiple knowledge systems are integrated to ensure sustainable adaptation at all levels.<br><br>It stakes out the direction for further studies on linkages between sustainability and adaptation. |
| 5. | Reindeer herders, active to a varying degree in reindeer husbandry or retired practitioners | Co-production | Aim at understanding, from the reindeer herders' own perspective, the consequences of the combined effects of people, animals, and climate change that shape their pastoral landscape in northern Fennoscandia as well as the herders' adaptive capacity to react to these changes by using their animals to modify the structure and function of the landscape | Empowerment and capacity building | Contact with the district before the onset, either personal or collaborational | Vegetation classification map, Statistics, Data sources for reindeer numbers | Focus groups, semi structured interviews | Yes, follows the ethical guidelines | Scientists and indigenous scientists | Scientists and indigenous scientists along with participants | Scientists and indigenous scientists along with participants | Scientists and indigenous scientists along with participants | Scientists and indigenous scientists | Documentation and empowerment of reindeer herder knowledge, which can potentially be used in management. |
| 6. | Long-term Village of Wainwright residents | Co-production | A summary of key concepts in visual theory that makes the case for context rich images as tools to help overcome information biases plaguing transdisciplinary governance efforts<br><br>Practical recommendations for reporting techniques in public sector environmental management, and for future studies attempting to understand the potential of MEB and visual reporting approaches. | Novel methods and tools | Iterative, participatory process in partnership with the local tribal council and local leader | | Semi-structured Interviews, visual images, questionnaire | Yes | Scientists | Scientists and participants | Scientists and participants | Scientists and participants | Scientists | A model to improve communication between place/based information beyond potential of basic text that can be beneficial for management. This is a model for knowledge brokering and communication that facilitate the transmission of place-based knowledge for decision-making |
| 7. | Community members, elders, youth indigenous researchers | Co-production | Implement a sustainable collaborative CBEM program of the George River watershed, as requested by the community. Study water quality and environmental change at the watershed scale. | Ecosystem based adaptation | Request from scientists of co-production to indigenous research | Science camp with sampling, remote sensing, | Brainstorm, science camp, collaborative | Yes | Scientists | Scientists and indigenous and | Scientists and indigenous and | Scientists and indigenous and | Scientists and indigenous and | Recommendations for local success in CBM. Colearning and engaging in common activities a CBM entails.<br>Improved culture capacity for individuals that are unfamiliar with the local culture. Increased relevance for science for locals, researchers and |

| | | | | | | | | | | | | | | |
|---|---|---|---|---|---|---|---|---|---|---|---|---|---|---|
| | | | | | institutions and tribe | interactive multimedia. Photos, GPS and environmental data | CBEM program, interviews | | | participants | participants | participants | participants | locals share same skills which can contribute to these groups to become allies instead of aliens |
| 8. | Indigenous and non-Indigenous community members | Co-production | A systematic, transparent, and replicable framework for how LEK, TEK, and SK can be brought together to inform wildlife status assessments. The study draws on previous experiences collaboratively studying muskox and caribou health in Nunavut and the Northwest Territories to illustrate how different approaches to monitoring, including hunter-based or harvest-based sampling and qualitative methods can be integrated with conventional scientific monitoring in the proposed assessment framework. | Novel methods and tools | Inspired by a scientific research project on salmon based on SK | Aerial survey, live-animal captures | Hunter-based sampling: biological samples and hunters' observations, interviews | yes | Scientists | Scientists and indigenous and participants | Scientists and indigenous and participants | Scientists and indigenous and participants s | Scientists and indigenous and participants | Bridging different knowledge types, and thus, different data sets, allows the framework to connect information from separate monitoring initiatives. Indicator-based assessment framework with a traffic light approach when including sampling approaches and knowledge types that provides a clear and intuitive output that can be applied by co-management boards |
| 9. | Inuit artists | Cross-fertilization | assess how Inuit artists themselves communicate the meanings and symbolism of their work. I examine artists' insights about the sea ice and climate change in their artworks and explore how these insights contribute to efforts to bridge Indigenous and Western scientific knowledge systems. | Novel methods and tools | Trust building through prolonged time spent in the village. Community outreach through radio. House visits. Meeting s with stakeholders. | Community researchers hired to communicate and translate. | semi structured interviews and dialogue, one-on-one art-making projects. | Yes | Scientists | Scientists | Scientists | Scientists and community researcher | Scientists | The artists showcased in this study reflect experiences of disappearing sea ice and climate change in their artworks. Beliefs, including values and emotional responses to sea ice and climate change, are also illuminated by Inuit art. Through art and artistic process, knowledge can be bridged between Inuit and Western scientific knowledge systems; likewise, it can be bridged between Elders and youth. In both cases, artworks and artistic processes create a platform to connect with the many dimensions of knowledge, including content, values and beliefs, emotions, and sensory experience. |
| 10. | Fishers and agency staff | Cross-fertilization | Is there evidence to suggest that nearshore rockfishes in Alaska are experiencing reduced abundance, demographic changes, or other ecological shifts? (2) Do fishermen and agency staff have concerns about the continued viability of rockfish fisheries? If so, what is the nature of these concerns? | Ecosystem based adaptation | Potential research participants were initially identified through key contacts. Snowball sampling. Invitation letter to the tribe for participation in the project. | ADF&G harvest data. Existing technical reports and other publications detailing changes in rockfish | 38 in-person semi-structured interviews. Open-ended interview | Yes | Scientists | Scientists | Scientists and participants s | Scientists | Scientists and participants s | Convey a plurality of perceptions, enrich our understanding about rockfish fisheries, and provide a basis for further inquiry. Generate a shared understanding of patterns and drivers of change in rockfish populations and fisheries and lends support to place-based, community-driven stewardship of nearshore rockfish fisheries. |

| | | | | | | | | | | | | | | |
|---|---|---|---|---|---|---|---|---|---|---|---|---|---|---|
| | | | | | Community meetings. | fisheries and management | | | | | | | | |
| 11. | Horse and cattle pastoralists | Cross-fertilization | The Sakha's relationship with Alaas is demonstrated. The reader is invited to understand this inductively, chronicling the relationship of Sakha people with alaas via historical changes and personal testimonies. Through this on- the- ground witness, longitudinal ethnography is used to reveal a culture's vernacular knowledge of human- environment interactions, in this case, Sakha and Alaas. The centrality of place attachment, cultural identity, and the overall sentience of the world in the context of unprecedented climate change is illustrated. | Climate risk perceptions | Longitudinal ethnography | - | Interviews, focus groups and oral histories, participation in daily life | Co-created research foci and field plans, mentored research assistants, collectively written community materials, and collaboration with Sakha scientists | Scientists | Scientists | Scientists | Scientists | Scientists | Both the Indigenous knowledge and scientific knowledge of alaas, taken together, give the most comprehensive understanding how alaas are central to Sakha identity, and provide a pathway to policy prescriptions that are more comprehensive, ethical, and rights- holder focused. |
| 12. | Horse and cattle pastoralists | Cross-fertilization | The first is how inhabitants continue to attribute change to a long-disproven driver, de facto perpetuating a cultural myth. The second is how inhabitants expressed starkly contrasting perceptions of change, specifically from 'changes are unprecedented and dramatic,' to 'nothing has changed, all is as it was before.' For both I argue the importance of using a cultural framing founded in a people's vernacular knowledge system with a focus on historical precedence for the former and on sacred belief for the latter. | Climate risk perceptions | Longitudinal ethnography | - | interviews, focus groups, and oral histories, participating in daily life | co-created research foci and field plans, mentored research assistants, collectively written community materials, and collaborated with Sakha scientists. | Scientists | Scientists | Scientists | Scientists | Scientists | Knowledge documentation |
| 13. | Inuit knowledge holders | Cross Fertilization | this study considers both Inuit and western scientific<br>knowledge of marine mammal distribution to understand the potential impact of ship-source underwater noise for narwhal, beluga and bowhead whales in Tallurutiup Imanga (TI) National Marine Conservation Area (NMCA) | Novel methods tools | Communitt organizations as research participant recognizers | Vessel traffic and ship noise data. Population data. | participatory mapping workshops of mammal observations and semi-structured interviews. | yes | Scientists | Scientists | Scientists | Scientists | Scientists | Inform decision-making related to ship traffic in the TI NMCA, and reiterates the importance of including both Inuit knowledge and western science. Helps to support decision- and policy-makers in their plans to manage vessels in TI NMCA, with a focus on the potential impacts of underwater noise from ships. |

| | | | | | | | | | | | | | | |
|---|---|---|---|---|---|---|---|---|---|---|---|---|---|---|
| | | | | | | | Inuit knowledge on cetacean | | | | | | | |
| 14. | Inuit commercial fishers. Inuit elder | Cross fertilization | assess whether the isotopic niche of anadromous Arctic Char shifted over a ~ 30-year time period (1987–2016) in the Kitikmeot marine region of Nunavut; (2) determine whether isotopic niche changes were aligned with climatic changes over this time period; and (3) explore the implications of reported climatic and ecological changes for Arctic Char fisheries, | Climate impacts | Community consultation at project onset. community throughout the project | Arctic char samples. Length, tissue. Prey samples. Zoplancton. Lab analysis. Climatic proxys. | semi-structured interviews with key informants. community engagement activities | Yes | Scientists | Scientists | Scientists | Scientists | Scientists | Novel set of transdisciplinary approaches were used to bridge different pieces of evidence into a cohesive, yet nuanced, story of ecological changes and their social-ecological implications.<br>Such community-collaborative research efforts will be critical to develop appropriate adaptation and management strategies and build resilience in responding to rapid shifts.<br>Lessons learned for future research : include the locals in the design process. |
| 15. | Native Alaskan representatives and 8 resource managers | Cross fertilixation | model the current habitat suitability and potential future climatic suitability of elodea in Alaska using an ensemble of SDM algorithms, in concert with global and Alaska-specific species occurrence records and environmental predictors, (2) assess the vulnerability of two important fish subsistence sources defined by Native Alaskans (Chinook salmon and whitefish) to elodea invasion across the state based on the six geographically distinct cultural/linguistic domains, and (3) discuss the utility of integrative modeling approaches as a decision-support tool for resource management. | Climate impacts | Summit (conference ) participation | Chinook salmon and whitefish spawning and rearing site data. Climate data | Interview and observational data. Existing data on subsistence practices in Alaska. | Ethical clearence | Scientists | Scientists | Scientists | Scientists | Scientists | The integrative modeling approach holds great utility for reframing how land management agencies engage in invasive species mapping and may further facilitate more holistic approaches to risk assessments. By incorporating the concerns of potentially affected resource-dependent communities, critical conservation targets, and disturbance drivers, such efforts can collectively identify management goals in a more inclusive and collaborative fashion |
| 16. | Hunters in Utqiaġvik | Co-production | Our goal was to document detailed IK for these species because they are important for subsistence in many Alaskan communities, and there is an urgent need to better understand habitat requirements to inform conservation measures in the face of climate change. | Climate impacts | | | semi-directed interviews with hunters | yes | Scientists | Scientists | Scientists | Scientists | Scientists and stakeholders | The IK shared provides extensive information on the habitat use of bearded, ringed, and spotted seals, along with information on foraging activity and dive behavior in the region around Utqiaġvik.<br>Additionally, the IK documented here has provided information at finer spatiotemporal scales than those typically associated with telemetry studies (e.g., use of currents as circuits). The IK shared in Utqiaġvik highlights the importance of inland water bodies and nearshore habitat for foraging, which is critical when considering habitat for conservation and management of these species (e.g., locations of roads or other infrastructure). |

| | | | | | | | | | | | | | | |
|---|---|---|---|---|---|---|---|---|---|---|---|---|---|---|
| | | | | | | | | | | | | | | IK remains invaluable to understand the impacts of climate change on ice-associated species, and the impacts to the communities that rely on them. |

## List of papers in the review:

1. Falardeau, M., et al. (2018). "A novel approach for co-producing positive scenarios that explore agency: case study from the Canadian Arctic." Sustainability Science **14**(1): 205-220.
2. Mellegård, V. and W. J. Boonstra (2020). "Craftsmanship as a Carrier of Indigenous and Local Ecological Knowledge: Photographic Insights from Sámi Duodji and Archipelago Fishing." Society & Natural Resources 33(10): 1252-1272.
3. Burt, J. M., et al. (2020). "Enabling coexistence: Navigating predator-induced regime shifts in human-ocean systems." People and Nature 2(3): 557-574.
4. Risvoll, C., et al. (2022). "Falling between the Cracks of the Governing Systems: Risk and Uncertainty in Pastoralism in Northern Norway." Weather, Climate, and Society 14(1): 191-204.
5. Horstkotte, T., et al. (2017). "Human-animal agency in reindeer management: Sami herders' perspectives on vegetation dynamics under climate change." Ecosphere 8(9).
6. Curry, T. and E. D. S. Lopez (2020). "Images as Information: Context-Rich Images and the Communication of Place-Based Information Through Increased Representation in Environmental Governance." Frontiers in Communication 5.
7. Gérin-Lajoie, J., et al. (2018). "IMALIRIJIIT: a community-based environmental monitoring program in the George River watershed, Nunavik, Canada." Écoscience 25(4): 381-399
8. Peacock, S. J., et al. (2020). "Linking co-monitoring to co-management: bringing together local, traditional, and scientific knowledge in a wildlife status assessment framework." Arctic Science 6(3): 247-266.
9. Rathwell, K. J. (2020). ""She is Transforming:" Inuit Artworks Reflect a Cultural Response to Arctic Sea Ice and Climate Change." Arctic 73(1): 67-80.
10. Gordon, J. Y., et al. (2022). "Bridging expert knowledge and fishery data to examine changes in nearshore rockfish fisheries in the Gulf of Alaska over fifty years." Fisheries Research 252: 106333.
11. Crate, S. A. (2022). "Sakha and Alaas: Place Attachment and Cultural Identity in a Time of Climate Change." Anthropology and Humanism 47(1): 20-38.
12. Crate, S. (2021). "Using Cultural Framings to Disentangle Viliui Sakha Perceptions, Beliefs, and Historical Trauma in the Face of Climate Change." Sibirica-Interdisciplinary Journal of Siberian Studies 20(3): 46-74.
13. Kochanowicz, Z., et al. (2021). "Using western science and Inuit knowledge to model ship-source noise exposure for cetaceans (marine mammals) in Tallurutiup Imanga (Lancaster Sound), Nunavut, Canada." Marine Policy 130: 104557.
14. Falardeau, M., et al. (2022). "Biophysical indicators and Indigenous and Local Knowledge reveal climatic and ecological shifts with implications for Arctic Char fisheries." Global Environmental Change-Human and Policy Dimensions 74.
15. Luizza, M. W., et al. (2016). "Integrating subsistence practice and species distribution modeling: assessing invasive elodea's potential impact on Native Alaskan subsistence of Chinook salmon and whitefish." Environmental Management 58(1): 144-163.
16. Gryba, R., et al. (2021). "Indigenous Knowledge of bearded seal (Erignathus barbatus), ringed seal (Pusa hispida), and spotted seal (Phoca largha) behaviour and habitat use near Utqiaġvik, Alaska, USA." Arctic Science **7**(4): 832-858.
